# Contribution to Understanding the Mechanisms Involved in Biofilm Formation, Tolerance and Control

**DOI:** 10.3390/ijms24119475

**Published:** 2023-05-30

**Authors:** Lúcia Chaves Simões, Manuel Simões

**Affiliations:** 1 CEB-Centre of Biological Engineering, University of Minho, Campus de Gualtar, 4710-057 Braga, Portugal; 2 LABBELS—Associate Laboratory, 4710-057 Braga, Portugal; 3 LEPABE, Department of Chemical Engineering, Faculty of Engineering, University of Porto, Rua Dr. Roberto Frias, s/n, 4200-465 Porto, Portugal; 4 ALiCE—Associate Laboratory in Chemical Engineering, Faculty of Engineering, University of Porto, Rua Dr. Roberto Frias, 4200-465 Porto, Portugal

**Keywords:** antimicrobial therapy, combinatorial therapy, biofilm formation, biofilm prevention and control, resistance

## Abstract

Biofilms constitute a protected mode of growth that allows the colonizing microbial cells to survive in hostile environments, even when an antimicrobial agent is present. The scientific community has come to understand many things about the growth dynamics and behavior of microbial biofilms. It is now accepted that biofilm formation is a multifactorial process that starts with the adhesion of individual cells and (auto-)coaggregates of cells to a surface. Then, attached cells grow, reproduce and secrete insoluble extracellular polymeric substances. As the biofilm matures, biofilm detachment and growth processes come into balance, such that the total amount of biomass on the surface remains approximately constant in time. The detached cells retain the phenotype of the biofilm cells, which facilitates the colonization of neighboring surfaces. The most common practice to eliminate unwanted biofilms is the application of antimicrobial agents. However, conventional antimicrobial agents often show inefficacy in the control of biofilms. Much remains to be understood in the biofilm formation process and in the development of effective strategies for biofilm prevention and control. The articles contained in this Special Issue deal with biofilms of some important bacteria (including pathogens such as *Escherichia coli*, *Pseudomonas aeruginosa* and *Staphylococcus aureus*) and fungi (*Candida tropicalis*), providing novel insights into their formation mechanisms and implications, together with novel methods (e.g., use of chemical conjugates and combinations of molecules) that can be used to disrupt the biofilm structure and kill the colonizing cells.

It is a natural tendency of microorganisms to attach to surfaces, multiply and embed themselves in a matrix of extracellular polymeric substances, resulting in biofilms. Biofilms are ubiquitous and versatile. Intentional and unintentional biofilms concern a broad range of areas, attracting special attention in the industrial and medical areas [1,2]. Biofilms form through several mechanisms through which microorganisms can come into closer contact with a biotic or abiotic surface, attach firmly to it, promote cell–cell interactions and grow as a complex structure where interspecific and intraspecific interactions are established among different (micro)organisms. In biofilms, the colonizing microorganisms create their own microniche and benefit from such a sessile association (i.e., excretion of metabolic compounds under the limitation of other growth factors; increased absorption of nutrients by the matrix of extracellular polymeric substances; increased availability of nutrients through uptake from bulk media; interspecies support within nutritional chains; cellular communication through quorum sensing mechanisms; protection against desiccation and other forms of environmental stress) [3].

Even if much is known about the mechanisms of biofilm formation, the scientific community has not established a generic and irrefutable mechanistic assessment. A revisited biofilm formation mechanism/life cycle has been recently published, but it exclusively focused on the sessile structure of heterotrophic vegetative bacteria [4]. It remains unknown whether other heterotrophic microorganisms, non-vegetative microbial structures (i.e., cysts and spores) and autotrophic microorganisms will follow the same steps enumerated in the revisited mechanism. It seems obvious that generalizations cannot be made when studying biofilms. In fact, such an approach would contradict the evolutionary perspective of microbiology, where microbial adaptation and stochastic switching events cannot be disregarded.

The Special Issue “Mechanisms in Biofilm Formation, Tolerance and Control” is composed of one review article and ten articles of original research, which contribute to expanding the current knowledge of biofilm science. The Special Issue is particularly focused on the medical area, where an authoritative review by Afonso et al. [2] describes the involvement of biofilms in diabetic foot ulcers as well as the clinical implications of the presence of biofilms. The concept of biofilms in human health and disease is now widely accepted as a cause of chronic infection, where biofilms show remarkable tolerance to antibiotic therapy and the host immune response. This study further reviews the current and promising alternative therapeutic strategies to control diabetic foot ulcers. An algorithm for the management of diabetic foot ulcers considering biofilm detection and treatment is proposed.

Original approaches to studying biofilm formation were published. In particular, Spengler et al. [5] used single-cell force spectroscopy and genetically modified *Staphylococcus aureus* cells to provide insights into the adhesion process of the pathogen to abiotic surfaces of varying wettability. Their study provided results of potential relevance to designing new models of bacterial adhesion, where the authors reported that *S. aureus* utilizes different cell wall molecules and interaction mechanisms when binding to hydrophobic and hydrophilic surfaces. Sedghizadeh et al. [6] utilized a real-time impedance-based assay to study the growth of *Staphylococcus aureus* biofilms over time. Helbig et al. [7] investigated the microbial adhesion within the human oral cavity on a low-surface-energy material (perfluorpolyether) with different texture types, feature sizes and graded distances. These authors found that adhesion to the oral environment cannot be controlled by structural means. Chen et al. [8] found that boron (an essential element for autoinducer-2 synthesis of quorum sensing systems) promoted biofilm formation by upregulating the expression levels of biofilm-related genes, improving the autoinducer-2 activity and increasing the secretion of extracellular polymeric substances by *Escherichia coli*.

Some studies further sought to prevent biofilm formation, while others aimed to develop antimicrobial agents to treat existing biofilms, or attempted to disrupt the polymeric ties that bind the biofilms together. As an example, Artini et al. [9] evaluated the activity of the metalloprotease serratiopeptidase in impairing virulence-related properties in *Pseudomonas aeruginosa*. The enzyme was able to impair bacterial adhesion to abiotic surfaces as well as the adhesion and invasion of eukaryotic cells. Sedghizadeh et al. [6] assessed biofilm inhibition by novel conjugates (bisphosphonate-conjugated versions of ciprofloxacin, moxifloxacin, sitafloxacin and nemonoxacin). The conjugates demonstrated varying action depending on the specific antibiotic used for conjugation, the type of bisphosphonate moiety, the chemical conjugation scheme and the presence or absence of hydroxyapatite. The antimicrobial activity of the parent antibiotic in the presence or absence of hydroxyapatite was retained. The novel antifungal agent arylamidine T-2307 demonstrated relevant antifungal and antibiofilm activity with low toxicity both in vitro and in vivo [10].

While this Special Issue provides pioneer results on biofilm science and technology, it is still clear that the significance of biofilms is not a well-understood phenomenon because of fundamental evolutionary aspects that allow microorganisms to adapt and persist in specific environments, a lack of direct observation of biofilms in their environment and a lack of research using model systems that closely simulate the environment where the biopellicle is present. Inhibition of biofilm formation and control of established biofilms using the current approaches remain virtually impossible procedures. By shedding light on the mechanisms involved in biofilm formation and persistence, we hope to contribute to the development of effective strategies for the prevention and control of biofilms.

## Data Availability

Data sharing not applicable.
